# Psychometric properties of self-report measures of eating disorder cognitions: a systematic review

**DOI:** 10.1186/s40337-023-00947-0

**Published:** 2023-12-20

**Authors:** Amaani H. Hatoum, Amy L. Burton, Sophie L. Berry, Maree J. Abbott

**Affiliations:** 1 School of Psychology, The University of Sydney, Level 2, 94 Mallet Street, Camperdown, Sydney, NSW 2006 Australia; 2 Graduate School of Health, University of Technology Sydney, Sydney, NSW Australia

**Keywords:** Eating disorders, Self-report, Cognitive, Psychometric, Systematic review

## Abstract

**Background:**

Although eating disorder (ED) models display some differences in theory and treatment approach, cognitive-behavioural, schema-focused, and disorder-specific models all highlight the fundamental nature of cognitions as key factors in ED development and maintenance processes. As such, it is vital that ED cognitions continue to be assessed and monitored as therapeutic targets and treatment outcomes as well as being examined as constructs in empirical research. This review aimed to systematically identify and evaluate the psychometric properties of existing self-report measures of ED cognitions.

**Methods:**

A systematic review protocol was registered using the international prospective register of systematic reviews (PROSPERO; CRD42023440840). Included studies described the development, validation and/or the psychometric evaluation of a measure (or subscale) that was specifically developed to solely assess ED cognitions (that is thoughts, expectations, assumptions, or beliefs), in English-speaking, adult populations. The search was conducted using three electronic databases: PsycINFO, MedLine, and Embase. Two independent reviewers conducted screening, selection and evaluation of the psychometric properties of relevant measures using a standardised, well-established quality appraisal tool.

**Results:**

Of the initial search of 7581 potential studies, 59 met inclusion criteria and described the psychometric evaluation of 31 measures (or subscales) of ED cognitions. The findings from the current review indicate that of the included measures, none currently meet all nine criteria of adequate psychometric properties. The Eating Beliefs Questionnaire (EBQ; and EBQ-18), and the Eating Disorder Inventory Body Dissatisfaction subscale (EDI [BD]) currently possess the most evidence supporting their validity, reliability, and clinical utility.

**Conclusions:**

The findings of the current systematic review provide guidance for future researchers to focus efforts on improving evidence for the validity, reliability and utility of self-report measures of ED cognitions. Overall, the present study has provided a detailed and systematic evaluation to support researchers and clinicians in future selection of measures of ED cognitions dependent on the specific aims of their research and treatment.

## Abstract (plain language summary)

Theoretical and empirical research suggests that eating disorder (ED) cognitions (that is, thoughts, expectations, assumptions, and beliefs) are important factors contributing to the development and maintenance of EDs. As such, it is important to continue to assess and monitor these in research and in clinical practice. Valid, reliable and useful assessment tools are critical in this process. This systematic review aimed to identify and evaluate the psychometric properties of existing self-report measures of ED cognitions. Included studies described the development, validation and/or the psychometric evaluation of a measure specifically developed to solely assess ED cognitions. Three electronic databases were searched, and studies screened and selected by two independent reviewers. The included studies and self-report measured were evaluated using a standardised tool to evaluate their psychometric properties. Fifty-nine studies were included, that identified a total of 31 measures (or subscales) assessing ED cognitions. The findings from the current review indicate that none of the included measures currently meet all nine criteria of adequate psychometric properties. The EBQ, EBQ-18, and EDI-III (BD) currently have the most evidence in support of their psychometric properties. This study provides direction for future researchers to focus efforts on improving evidence for the validity, reliability and utility of these self-report measures. Overall, we provided information to support researchers and clinicians in future selection of measures of ED cognitions.

## Introduction

The prevalence and impact of eating disorders (EDs) continues to increase worldwide [2, 15, 32, 73]. Currently, the Diagnostic and Statistical Manual of Mental Disorders [1] outlines the criteria for several disorders, including but not limited to anorexia nervosa (AN), bulimia nervosa (BN), binge eating disorder (BED), and other specified feeding and eating disorder (OSFED). The functional impact these disorders has been well established, with existing literature highlighting that EDs are associated with higher medical and psychiatric comorbidity, reduced quality of life and increased risk of mortality [2, 90, 91]. Despite the existence of various treatment models, and some evidence of increased treatment seeking [73], treatment outcomes are varied, and efficacy typically only achieves modest gains [92, 93]. As such, examining the key components implicated in both the development and maintenance of EDs remains critical.

Theoretical ED models emphasise the importance of ED relevant cognitions as both development and maintenance factors [21, 27, 94]. Cognitions implicated in the development and maintenance of EDs include thoughts, expectations, assumptions, and conditional and unconditional beliefs. From a transdiagnostic perspective, Fairburn et al.’s [27] cognitive-behavioural model of EDs highlights the role of over-evaluation of eating, weight, shape and their control, core low self-esteem, as well as emphasising the role of unconditional, often pervasive, and negative views of the self (e.g., negative, self-referent beliefs [‘I am unlovable]). Core low self-esteem and negative self-beliefs contribute to an increasingly dysfunctional schema for self-evaluation and thereby contribute to the over-evaluation of eating, weight, shape, and their control. The over-evaluation of these features and their importance is often central in the initial emergence and maintenance of ED behaviours, such as restriction, purging and binge eating. Cognitive-behavioural therapy for EDs (CBT-E), the transdiagnostic treatment derived from this theory, has been shown to be an effective and efficacious treatment option for a range of ED diagnoses, in adults and adolescents [3, 24].

The schema-focussed model of EDs [94] clearly delineates the role of unconditional core beliefs or schema level representations in the development of different eating pathology. It suggests that in AN, schema compensation occurs to prevent experiencing negative affect, resulting in restrictive eating behaviours, whereas avoidance of negative affect generated by the activation of negative core beliefs results in more bulimic-type pathology [94]. Schema content may include conditional and unconditional beliefs about the self, others, or the world across several dimensions and schema domains. For example, Waller et al. describe the relationship between unconditional beliefs regarding the self (i.e., self-referent beliefs, such as ‘I am worthless’), others (e.g., ‘Others are judgemental and harsh’), and possible maladaptive schemas relating to these beliefs (e.g., ‘abandonment’ or ‘unrelenting standards’). Narrative and systematic reviews of schemas and schema therapy outcomes have indeed highlighted associations between pronounced early maladaptive schemas (EMS) and EDs [52, 70], and indicated that schema therapy interventions show some promise for treating complex eating presentations [52].

Additionally, several disorder specific models highlight the importance of core, self-referent beliefs, as well as other types of ED cognitions. The cognitive model of bulimia nervosa suggests that maladaptive, negative self-beliefs (e.g., ‘I am a failure’; [21] act as a predisposing factor, leading to increased negative automatic thoughts (NATs), which in turn serve to further reinforce these beliefs. The model also outlines the role of permissive, positive and negative beliefs about eating in the maintenance of binge eating and purging behaviours. These beliefs can be understood as types of meta-cognitive beliefs, that is, a set of higher order beliefs reflecting understanding, awareness and interpretation of one’s own thought processes [97]. For example, a positive meta-cognitive belief about eating might be ‘eating helps me to control my emotions’ [13]. An integrated cognitive-behavioural model of binge eating similarly identifies both the critical roles of core low self-esteem (negative core beliefs about the self) and eating beliefs (that is, meta-cognitive beliefs) in the development and maintenance of binge eating [11].

Models by Fairburn et al. [27] and Cooper et al. [21] both emphasise the role of NATs that reflect the over-evaluation of eating, weight, shape and its control in the maintenance of EDs. For example, negative thoughts about food and eating may be statements such as ‘I hate that I like to binge’, versus negative thoughts about weight and shape that may include content such as ‘I’ll gain a huge amount of weight’ or ‘I think my stomach is too big’. In accordance with previously described models [11, 21, 27], these negative and often automatic thoughts serve to maintain ED cycles by increasing negative affect or impacting emotional regulation, which in turn reinforce ED behaviours. These negative thoughts also ultimately reinforce more pervasive underlying cognitions (i.e., core, self-referent beliefs).

Although the aforementioned models display some differences in their cognitive emphasis (in both content and types of cognitions), each highlights the fundamental nature of ED cognitions as factors in ED developmental and maintenance processes. As such, it is vital that ED cognitions continue to be assessed and monitored as therapeutic targets and treatment outcomes. Both theoretical and empirical research has supported the idea of measuring ED ‘thoughts’ separately to behaviours, as well as assessing ED cognitions as a continuous construct that is often identified in sub-clinical or prodromal populations [53], where there is need to be proactive in prevention and early intervention. It is vital therefore to utilise assessment tools measuring ED cognitions that possess strong psychometric properties.

Previously, Burton et al. [9] conducted a systematic review of the psychometric properties of self-report measures relating specifically to binge-eating symptoms. Further, two recent systematic reviews reported on the available measures and facets relating to body image [48, 69]. However, each of these reviews did not provide a comprehensive summary relating to all EDs and related features more broadly. Another recent review summarised the instruments utilised in the assessment of EDs in adults [77], providing an overview of commonly used and recently developed measures of ED symptomatology. However, the focus of this review was only on frequently used and recently developed assessment measures. It did not specifically report on cognitive measures, that is, those with focus on ED beliefs, expectations, assumptions and thoughts. As such, several important existing instruments that have been developed to assess ED relevant cognitions were not reviewed. Some examples include the Eating Beliefs Questionnaire-18 (EBQ-18) [10], and the Eating Disorder Core Beliefs Questionnaire (ED-CBQ) [28]. Moreover, a thorough evaluation of the psychometric properties of the identified measures using a standardised, published tool for assessing their quality was not conducted, instead, the focus of the review was informative rather than evaluative [77].

Thus, to date, there has been no comprehensive assessment of the available self-report measures of ED cognitions or a thorough assessment of their psychometric properties. The aim of this review was to systematically identify and evaluate the psychometric properties of existing self-report measures of ED cognitions. The psychometric properties of these cognitive self-report measures will be evaluated using the appraisal of adequacy tool, developed by Terwee et al. [87]. This standardised tool guides quality appraisal by using nine quality criteria, including content validity, internal consistency, criterion validity, construct validity, reproducibility (agreement), reproducibility (reliability), responsiveness, floor or ceiling effects, and interpretability. This tool has previously been utilised in Burton et al. [9] review of self-report measures of binge-eating symptoms, as well as several other systematic reviews in other domains [59, 84, 101]. Using these criteria, this study intends to systematically summarise the available findings for cognitive ED self-report measurement tools, providing a comprehensive understanding of their psychometric properties, and guidance for researchers and clinicians in evaluating, comparing and utilising these measures.

## Method

### Search strategy

A systematic review protocol was registered using the international prospective register of systematic reviews (PROSPERO; CRD42023440840). The search strategy followed guidelines outlined in the Preferred Reporting Items for Systematic Reviews and Meta-analyses (PRISMA; [66]. Utilising guidelines for optimal database combinations for literature searches [8], the search was conducted using three electronic databases: PsycINFO, MedLine, and Embase. There were no limits to search based off publication period. Reference lists of all included studies were scanned to identify any additional, relevant publications. Searches were run again prior to final analysis on the 17/08/2023. To identify eligible studies, several combinations of keywords were used that related to EDs (e.g., “eating disorder”, “anorexia nervosa”, “bulimia nervosa”, “binge eating disorder”, etc.), self-report measures (e.g., “questionnaire”, “scale”, “tool”, “assessment”, “measure”, etc.), and psychometric properties (e.g., “psychometric”, “reliability”, “validity”, etc.). A comprehensive search was conducted, including search of titles, abstracts, and keywords, subject headings were mapped, and in some instances the explode function was utilised for expansion of relevant results. The full search strategy created for all three databases is a publicly available supplementary file included in the systematic review protocol registered on PROSPERO.

### Inclusion and exclusion criteria

The inclusion criteria were as follows:

1. The study had to describe the development, validation and/or the psychometric evaluation of a self-report measure of ED cognitions.
2. The measure (or subscale) was specifically developed to solely assess ED cognitions (that is thoughts, expectations, assumptions, or beliefs).
3. The measure was developed and administered in the English language, to native English speakers, and published in the English language in a peer review journal.
4. Utilised an adult population (17+, clinical or general) for development, evaluation or investigation purposes.

The exclusion criteria were as follows:

1. Non-psychometric studies (such as literature reviews, systematic reviews, or meta-analyses).
2. Measure (or subscale) was designed for purposes other than assessing ED cognitions (such as, as a screening or diagnostic tool, or that assesses behaviours or emotions).
3. Measure not in English, administered in English, or published in a non-English speaking country.
4. Utilised a child or adolescent population.
5. Book chapters, non-peer reviewed publications, published doctoral theses.

### Selection process

Articles were screened and selected by two independent reviewers (AH and SB). Using the identified databases, duplicates were identified and removed, and articles were screened by title and abstract for inclusion/exclusion by AH. During title and abstract screening process, a series of meetings were held with the four included authors, to provide consensus about the relevance of measures that were accessible during this stage. A similar process was utilised by a previous psychometric systematic review of body image [48]. Full texts of the remaining studies were obtained after the initial screening, then both reviewers analysed each text independently to establish the final texts to be included. Measures (and subscales) were also assessed for their relevance and eligibility at this stage of the screening process, if the measure was not available (at the item level) at the stage of title and abstract screening. Studies (and therefore measures) that required further scrutiny to assess whether they met inclusion criteria were included in full text screening in order to assess their relevance and eligibility at the subscale and item level. A final consensus meeting was conducted where all authors provided agreement as to the relevance of all included measures.^1^ The overall agreement between the two reviewers was 96.7%, which equates to an inter-rater agreement (Kappa) of κ = 0.93.

### Appraisal of quality

The psychometric properties of included studies were analysed using Terwee et al. [87] criteria of adequacy for measurement properties. This quality appraisal tool was designed to assess health status questionnaires and has been used in several previous systematic reviews [9, 59, 84, 101]. This tool assesses nine measurement properties, including (1) content validity, (2) internal consistency, (3) criterion validity, (4) construct validity, (5) reproducibility: agreement, (6) reproducibility: reliability, (7) responsiveness, (8) floor and ceiling effects, and (9) interpretability. See Table 1 for the definition and criteria of adequacy for each of the nine properties.

**Table 1:** Criteria of quality of psychometric properties [87]

Property	Definition	Criteria of adequacy
Content validity	The degree to which the content of an instrument is an adequate reflection of the construct to be measured	(+) A clear description is provided of the measurement aim, the target population, the concepts that are being measured, and the item selection AND target population and experts were involved in item selection (?) A clear description of above-mentioned aspects is lacking OR only target population involved OR doubtful design or method (−) No target population involvement (0) No information found on target population and experts’ involvement
Internal consistency	The degree which items are intercorrelated, thus measuring the same construct	(+) Factor analyses performed on adequate sample size (7 times the number of items) AND Cronbach’s alpha(s) or McDonald’s omega(s) between 0.70 and 0.95 for each scale (?) Cronbach’s alphas or McDonald’s omega(s) presented without factor analysis considered OR doubtful design or method (−) Cronbach’s alpha(s) or McDonald’s omega(s) < 0.70 or > 0.95 (0) No information found on internal consistency
Criterion validity	The degree to which the scores of an instrument are an adequate reflection of a ‘gold standard’	(+) Convincing arguments that gold standard is ‘‘gold’’ AND correlation with gold standard ≥ 0.70 (?) ≥ 0.70 correlation presented without convincing arguments that gold standard is ‘‘gold’’ OR doubtful design or method (−) Correlation with gold standard < 0.70 (0) No information found on criterion validity
Construct validity	The degree to which scores on a particular questionnaire relate (or are unrelated) to other measures in a manner that is consistent with theoretically derived hypotheses concerning the concepts that are being measured	(+) Explicitly tested for AND at least 75% of the results are in expected direction and size (e.g., reporting the correlation between two measures in the expected direction, or the expected lack of correlation) (?) Doubtful design or method (e.g., not explicitly tested) (−) Less than 75% of results as expected (0) No information found on construct validity
Reproducibility Agreement (test–retest)	The extent to which the scores on repeated measures are close to each other (absolute measurement error)	(+) Test–retest agreement r > .70 AND means and standard deviations are presented at both time points (?) > 0.70 correlation presented without means and standard deviations at both time points OR doubtful design or method (−) Test–retest agreement r < .70 (0) No information found on test–retest reliability
Reliability	The extent to which patients can be distinguished from each other, despite measurement errors (relative measurement error)	(+) T tests ICC or weighted Kappa > 0.70 (?) Doubtful design or method (e.g., time interval not mentioned or less valid measure then a Kappa used) (−) ICC or weighted Kappa < 0.70; (0) No information found on reliability
Responsiveness	The ability of an instrument to detect clinically important changes over time in the construct to be measured	(+) Treatment program outlined, and longitudinal expected changes presented AND > 75% of results are as expected OR RR > 1.96 OR AUC > 0.70 (?) Doubtful design or method (−) RR < 1.96 OR AUC < 0.70 (0) No information found on responsiveness
Floor and ceiling effects	The number of respondents who achieved the lowest or highest possible score	(+) < 15% of the respondents achieved the highest or lowest possible scores (?) Doubtful design or method (−) > 15% of the respondents achieved the highest or lowest possible scores (0) No information found on floor and ceiling effects
Interpretability	Degree to which one can assign qualitative meaning to an instrument’s quantitative scores or change in scores	(+) Mean and SD scores presented for at least four relevant subgroups of patients^*c*^ (?) Doubtful design or method (e.g., data provided on less than four subgroups) (0) No information found on interpretation

Adaptations made to supplement ‘Minimal important change’ (MIC): Criterion 5.1 (Reproducibility—Agreement) modified such that test–retest reliability is sufficient to receive a positive score. Criterion 7 (Responsiveness) modified such that MIC not utilised. Criterion 9 (Interpretability) modified such that MIC not needed to be defined for a positive score

*SDC *smallest detectable change, *LOA* limits of agreement, *ICC* Intraclass correlation, *AUC* area under the receiver operating characteristics curve, *RR* Responsiveness Ratio, *SD* standard deviation

^a^+  = positive rating; ? = indeterminate rating;—= negative rating; 0 = no information available

^b^Doubtful design or method = lacking a clear description of the design or methods of the study, sample size smaller than 50 subjects, or any important methodological weakness in the design or execution of the study

^c^Terwee et al. (2007) have used the term ‘patients’ in this table given the original application of these criteria to medical populations. More recently, the quality criteria have been employed to assess measures relevant to a variety of populations, including clinical, non-clinical, and normative samples. Despite not including medical samples in the present review, we have retained the term ‘patients’ here in order to present Terwee et al.’s original criteria

Printed with permission from the publisher: Elsevier

Criteria were given the following evaluative ratings; positive (+), intermediate (?), negative (−), no information available (0). Intermediate ratings may be given if there are serious doubts about study methodology, as per the guidelines for utilising these criteria [87]. It is essential to consider the methodological quality of included studies when assigning a rating, as those with low methodological quality will have a greater likelihood of reporting biased results. Further, the criteria for internal consistency allowed studies to consider the results of past factor analyses, or item response theory (IRT) analyses, when conducting a Cronbach alpha (i.e., as each study did not necessarily need to carry out a new factor analysis). Finally, Terwee et al. [87] indicate that all measurement properties are not necessarily equally important, thus, we followed the recommendation not to provide a summary or overall score.

Further, included studies were summarised by describing if they were a development study, any factor analyses performed, the study population, sample size, mean age and standard deviation, and the sex ratio (% females). Included measures (or subscales) were summarised by describing the construct or goal being measured, the number of items, response categories and any subscales or factors. Attempts were made to obtain missing or unclear information by contacting the authors of studies assessed for eligibility. Missing or unclear information that did not affect inclusion was still recorded (as either ‘?’ or Not Applicable [N/A]). In the absence of information from authors contacted, an assumption was maintained that if participants were described to be of ‘college’ or ‘university’ age, that they were a part of an adult sample (17+).

## Results

### Results of search strategy

**Fig. 1:**
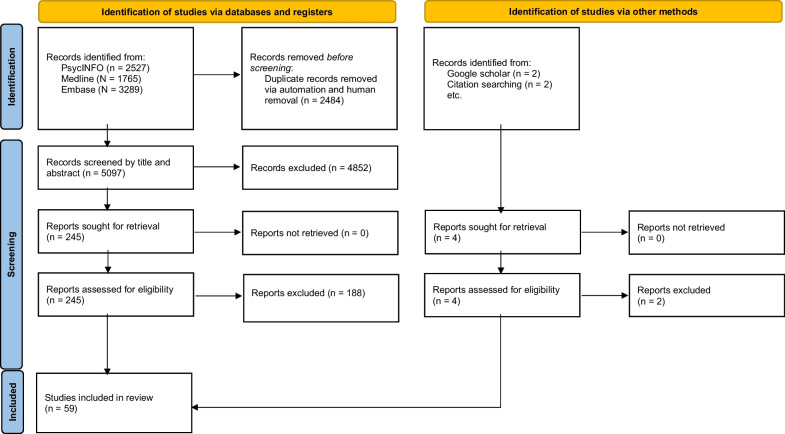
PRISMA diagram of study identification, screening and selection (PRISMA [66])

The initial search identified 7581 potential studies. After removal of 2484 duplicates, this resulted in 5097 potential studies, of which 57 were considered to have met the inclusion criteria. An additional two studies were identified by cross checking reference lists for articles of interest and searching google scholar. This resulted in a total of 59 included studies (see Table 2 for summary of included studies). The selection process is summarized in Fig. 1.

**Table 2:** Description of studies

Measure/study	Development Study	Factor analysis	Study population(s) and sample size(s)	Mean age (SD)	Sex ratio (% female)
*Beliefs About Appearance Questionnaire* *(BAAS)*					
Spangler and Stice [82]	No	PCA and CFA indicated a one-factor model	University Sample 1 = 462 University Sample 2 = 117	64% aged 18–21 Age range 17–29	53% 100%
*Bulimic Automatic Thoughts Test* *(BATT)*					
Franko and Zuroff [31]	No	No factor analysis performed	BN = 64 Depressed college = 20 Non-binge obese = 20 Control college = 20	25.20 (?) 20.60 (?) 20.60 (?) 20.60 (?)	? ? ? ?
*Body Checking Cognitions Scale* *(BCCS)*					
Mountford et al. [60]	Yes	PCA and CFA indicated a four-factor model: 1. Objective verification beliefs 2. Reassurance beliefs 3. Safety beliefs 4. Body control beliefs	Non-clinical = 180 Clinical ED = 84	22.40 (6.64) 28.30 (8.69)	100% 100%
*Bulimia Cognitive Distortions Scale* *(BCDS)*					
Bonifazi et al. [7]	No	No factor analysis performed	BN = 15 Restrained eaters = 15 Control = 15	20.10 (2.20) 19.50 (2.60) 19.40 (1.30)	100% 100% 100%
Schulman et al. [80]	Yes	EFA indicated two-factor model: 1. Cognitive distortions associated with automatic eating behaviours 2. Cognitive distortions associated with physical appearance	BN = 55 Control = 55	24.50 (?) 22.60 (?)	100% 100%
*Bulimic Thoughts Questionnaire* *(BTQ)*					
Phelan [68]	Yes	CFA indicated a three-factor model: 1. Self-schema 2. Self-efficacy 3. Salient beliefs	BN = 31 Obese = 20 Control = 22	N/A (‘college’)	100%
*Eating Beliefs Questionnaire* *(EBQ)*					
Burton et al. [12]	No	CFA indicated a two-factor model: 1. Positive beliefs about eating 2. Negative Beliefs about eating	Community = 290 University = 283 BE = 76 Obese = 120	27.54 (9.57) 20.23 (4.80) 35.97 (17.68) 42.32 (9.51)	67.9% 52.3% 100% 58%
Burton et al. [14]	No	No factor analysis performed	BN = 38 BED = 36 Control = 114	23.08 (4.45) 49.72 (16.35) 29.12 (10.34)	100% 100% 70.2%
*Eating Beliefs Questionnaire 18* *(EBQ-18)*					
Burton and Abbott [10]	No	EFA indicated and CFA supported a three-factor model: 1. Positive beliefs about eating 2. Negative beliefs about eating 3. Permissive beliefs about eating	University = 907	20.38 (4.88)	72%
Burton et al. [13]	No	CFA supported a three-factor model as in Burton and Abbott [10]	Total sample = 688	25.38 (11.82)	63.1%
*Eating Disorder Beliefs Questionnaire* *(EDBQ)*					
Cooper et al. [18]	Yes	PCA indicated a four-factor model: 1. Negative self-beliefs 2. Weight and shape as a means of acceptance by others 3. Weight and shape as a means to self-acceptance 4. Control over eating	Study 1: Non-clinical = 249	20.90 (?)	100%
Bergin and Wade [6]	No	CFA supported four-factor model as in Cooper et al. [18]	Non-clinical = 298 BN = 44	24.00 (9.65) 27.00 (7.76)	100% 97.7%
*Eating Disorder Core Beliefs Questionnaire* *(ED-CBQ)*					
Fairchild and Cooper [28]	Yes	EFA indicated five-factor model: 1. Self-loathing 2. Unassertive/inhibited 3. High standards for self 4. Demanding/in need of help and support 5. Abandoned/deprived	Non-clinical = 500	26.25 (8.70)	100%
*Eating Disorder Core Beliefs Questionnaire Revised* *(ED-CBQ-R)*					
Hatoum et al. [40]	No	CFA indicated four-factor model: 1. Self-loathing 2. Unassertive/inhibited 3. Demanding/in need of help and support 4. Abandoned/deprived	Non-clinical = 763	19.21 (3.21)	71%
Hatoum et al. [41]	No	CFA supported four-factor model as in Hatoum et al. [40]	Non-clinical = 283	20.23 (4.80)	52.3%
*Eating Disorder Inventory—Body Dissatisfaction Subscale* *(EDI [BD])*					
Garner et al. [36]	Yes	No factor analysis performed	AN Restrictors = 48 AN Bulimics = 65 Non-clinical 1 = 577 Non-clinical 2 = 166 Normal weight bulimic = 195 Obese = 44 Formerly obese = 52 Recovered anorexic = 17	21.00 (?) 22.40 (?) 19.90 (?) 20.30 (?) 20.80 (?) 32.50 (?) 36.70 (?) 23.90 (?)	100% 100% 100% 0% 100% 100% 100% ?
Cooper et al. [19]	No	No factor analysis performed	Psychiatric outpatients = 27	N/A (Range 17–39)	100%
Gross et al. [38]	No	No factor analysis performed	Bulimia = 82	24.30 (?)	100%
Raciti and Norcross [71]	No	PCA supported the hypothesised eight-factor model (including the *body dissatisfaction subscale*)	Non-clinical = 268	18.00 (0.78)	100%
Wear and Pratz [95]	No	No factor analysis performed	University = 70	N/A (‘University’)	75.7%
Welch et al. [96]	No	PCA indicated a three-factor model, not supporting the eight original EDI subscales (b*ody dissatisfaction subscale* not indicated as a separate factor/subscale)	University 1 = University 2 = Aerobic dancers = 142	20.00 (?) 21.00 (?) 26.00 (?)	? ? ?
*tt *et al*. (1990)*	No	No factor analysis performed	AN = 65 General psychiatric = 69 Bulimia = 66	23.00 (7.00) 31.00 (6.30) 26.10 (5.70)	100% 100% 100%
Klemchuk et al. [47]	No	PCA indicated a six-factor model, not supporting the eight original EDI subscales (*body dissatisfaction subscale* not indicated as a separate factor/subscale)	University 1 = 621 University 2 = 636 University 3 = 249	18.30 (1.00) 18.80 (0.70) 20.60 (0.80)	100% 100% 100%
Schaefer et al. [79]	No	EFA indicated a five-factor model, not supporting the eight original EDI subscales (including the *body dissatisfaction subscale*)	BN subsample = 48 EDNOS subsample = 17 Total ED sample = 79	24.40 (4.00) 29.00 (7.77) ? (?)	100% 100% 100%
*Eating Disorder Inventory II*—*Body Dissatisfaction Subscale* *(EDI-II [BD])*					
Tasca et al. [86]	No	CFAs supported a second-order two factor structure for the original EDI scales in the BED sample but not the BN sample CFAs did not support the hypothesised two-factor structure in either sample	BED = 144 BN Purging = 152	41.97 (12.35) 29.39 (8.80)	100% 100%
Spillane et al. [83]	No	CFA supported the hypothesised eight-factor structure, and provided evidence for invariance of *body dissatisfaction subscale* across gender	University 1 = 215 University 2 = 214	18.48 (1.07) 18.83 (1.15)	100% 0%
Reilly et al. [72]	No	No factor analysis performed	University = 529	N/A (‘University’)	55.4%
*Eating Disorder Inventory III*—*Body Dissatisfaction Subscale* *(EDI-III [BD])*					
Kashubeck-West et al. [46]	No	CFA did not support one-factor structure hypothesised for the *body dissatisfaction subscale* EFA suggested a two-factor model: 1. Stomach sizes 2. Thighs, hips, butt	University = 278	29.04 (9.35)	100%
Cordero et al. [22]	No	EFA using the ‘eating disorder risk composite’ subscales supported the hypothesised three-factor model: 1. Drive for thinness 2. Bulimia 3. Body dissatisfaction*	University = 248	20.30 (4.50)	97.6%
Stein et al. [85]	No	No factor analysis performed	University = 477	19.80 (2.40)	100%
Belon et al. [4]	No	CFA using the ‘eating disorder risk composite’ subscales supported the hypothesised three-factor model in the full sample: 1. Drive for thinness 2. Bulimia 3. Body dissatisfaction* Measurement invariance not supported in subsamples for the *body dissatisfaction subscale* (Caucasian and Hispanic subsamples)	University = 688	20.40 (3.50)	100%
Rothstein et al. [74]	No	CFA using the ‘eating disorder risk composite’ subscales supported the hypothesised three-factor model in European American subsample 1. Drive for thinness 2. Bulimia 3. Body dissatisfaction* A follow-up EFA using the ‘eating disorder risk composite’ subscales indicated a four-factor model in African American subsample: 1. Drive for thinness 2. Bulimia 3. Body dissatisfaction* 4. *Body satisfaction*	African American = 104 European American = 197	29.03 (11.37) 27.30 (9.82)	100%
Forbush et al. [30]	No	No factor analysis performed	University = 227	19.80 (3.00)	58.2%
*Eating Expectancy Inventory* *(EEI)*					
Williams-Kerver et al. [99]	No	CFA supported the hypothesised five-factor model: 1. Eating helps manage negative affect 2. Eating is pleasurable and useful as a reward 3. Eating leads to feeling out of control 4. Eating enhances cognitive competence 5. Eating alleviates boredom	Bariatric = 262	45.30 (12.80)	100%
*Functions of Binge Eating Scale* *(FBES)*					
O’Loghlen et al. [62]	Yes	EFA and CFA indicated an eight-factor model: 1. Self-protection 2. Compensatory eating 3. Hedonic hunger 4. Emotional regulation 5. Control 6. Self-punishment 7. Emotion expression 8. Numbness/dissociation	Non-clinical = 882	28.52 (9.55)	76.6%
*Irrational Food Beliefs Scale* *(IFBS)*					
Osberg et al. [64]	Yes	EFA indicated a two-factor model: 1. Irrational beliefs about food and eating 2. Rational beliefs about food and eating	University sample 1 = 139 University sample 2 = 58 University sample 3 = 301 Obese = 96	19.25 (2.56) N/A (‘college’) 19.54 (2.93) 49.50 (11.90)	81.3% 79.3% 68.1% 80.2%
*Interpersonal Outcome Expectancy for Thinness Scale* *(IOET)*					
Li et al. [49]	Yes	EFA indicated a one-factor model: 1. Positive interpersonal outcome expectancies for being thin	University sample 1 = 361 University sample 2 = 184	19.37 (1.56) 19.10 (1.55)	100% 100%
*Mizes Anorectic Cognitions Questionnaire* *(MACQ)*					
Mizes and Klesges [58]	Yes	PCA indicated a three-factor model: 1. Rigid weight and eating regulation 2. Weight and eating behaviour as the basis of approval from others 3. Self-esteem based off excessive self-control	Non-clinical = 205	N/A (‘college’)	48.8%
Mizes [54]	No	No factor analysis performed	Non-clinical = 205	N/A (‘college’)	48.8%
Mizes [55]	No	PCA supported three-factor model as in Mizes and Klesges [58]	Non-clinical = 100	18.50 (1.70)	100%
Mizes [56]	No	No factor analysis performed	BN = 15 AN = 8 Psychiatric Control = 11	‘18 + ’ (N/A)	86.7% 87.5% 90.9%
Bonifazi et al. [7]^a^	No	No factor analysis performed	BN = 15 Restrained eaters = 15 Control = 15	20.10 (2.20) 19.50 (2.60) 19.40 (1.30)	100%
*Mizes Anorectic Cognitions Questionnaire Brief* *(MACQ-B)*					
Osman et al. [65]	No	CFA indicated a three-factor model as in Mizes et al. [57]	Non-clinical = 290	20.63 (1.98)	66.6%
*Mizes Anorectic Cognitions Questionnaire Revised* *(MACQ-R)*					
Mizes et al. [57]	No	PCA supported three-factor model: 1. Self-control and self esteem 2. Weight and approval 3. Rigid weight regulation and fear of weight gain	AN = 44 BN = 97 AN (B/P) = 7 EDNOS = 57	25.90 (9.20)	97.1%
*Muscle Dysmorphia Inventory—Drive for Size Subscale* *(MDDI [DS])*					
Hildebrandt et al. [43]	Yes	PCA indicated a three-factor model: 1. Drive for size* 2. Appearance intolerance 3. Functional impairment	Weightlifters = 42 Weightlifters = 237	28.23 (8.07) 32.64 (12.37)	0% 0%
Compte et al. [16]	No	EFA and CFA supported the three-factor model as in Hildebrandt et al. [43]	Gay men = 715 Lesbian women = 404	35.40 (10.10) 31.60 (8.40)	0% 100%
Nagata et al. [61]	No	EFA and CFA supported the three-factor model as in Hildebrandt et al. [43]	Transgender men = 330	30.90 (9.80)	0%
*Perceived Benefits of Thinness Scale* *(PBTS)*					
Flatt et al. [29]	Yes	EFA and CFA indicated a one-factor model	Non-clinical = 3246	22.18 (5.31)	100%
*Sociocultural Attitudes Towards Appearance Questionnaire 4*—*Internalisation Thin/Low Body Fat subscale (SATAQ-4 [IT])*					
Schaefer et al. [75]	No	EFA and CFA indicated a five-factor model: 1. *Internalisation*—Thin/low body fat* 2. Internalization—Muscular/athletic 3. Pressures—Family 4. Pressures—Peers 5. Pressures—Media	Non-clinical = 859 Non-clinical = 440 Non-clinical = 304 Non-clinical = 349 Non-clinical = 362 Non-clinical = 271	20.17 (2.41) 18.71(1.01) 19.99 (1.69) 18.87 (1.61) 22.73 (2.82) 20.31 (1.75)	100% 100% 100% 100% 100% 0%
Schaefer et al. [76]	No	No factor analysis performed	Non-clinical = 787	20.17 (2.41)	100%
*Sociocultural Attitudes Towards Appearance Questionnaire 4 Revised-Internalisation Thin/Low Body Fat subscale* *(SATAQ-4R [IT])*					
Schaefer et al. [78]	No	EFA and CFA indicated a seven-factor model: 1. *Internalisation*—Thin/low body fat* 2. Internalization—Muscular/athletic 3. Internalisation: General attractiveness 4. Pressures—Family 5. Pressures—Peers 6. Pressures—significant others 7. Pressures—Media	Non-clinical = 566 Non-clinical = 548 Non-clinical = 133 Non-clinical = 290	20.53 (2.52) 20.55 (4.43) 19.59 (2.35) 20.84 (2.70)	100% 100% 100% 0%
Thompson et al. [88]	No	CFA indicated a bifactor model (indicating shared and unique constructs) for the 1. *SATAQ-4R -Internalisation*—Thin/low body fat subscale* 2. IBSS-R	University = 1114	20.54 (2.48)	100%
Convertino et al. [17]	No	CFA supported seven-factor model as in Schaefer et al. [78]	Non-clinical = 479 Non-clinical = 482	24.03 (3.76) 23.33 (3.69)	0% 100%
*Stirling Eating Disorders Scales* *Anorexic Dietary Cognitions subscale* *(SEDS [ADC])* *Bulimic Dietary Cognitions subscale* *(SEDS [BDC])* *Low Self-esteem subscale* *(SEDS [LSE])*					
Williams et al. [98]^*1*^	Yes	No factor analysis performed	AN = 38 BN = 36 Non-clinical control = 68	24.70 (5.30) 20.50 (6.10) 23.80 (4.90)	? ? ?
Openshaw and Waller [63]^*1*^	No	No factor analysis performed	BN = 40	28.40 (6.60)	100%
Gamble et al. [33]^*1*^	No	CFA did not support the original eight-factor model PCA indicated a five-factor solution, not matching the original eight SEDS subscales	Clinical ED = 241	26.8 (7.8)	?
*Testable Assumptions Questionnaire for Eating Disorders (TAQ-ED)*					
Hinrichsen et al. [44]	Yes	EFA indicated a three-factor model: 1. Dysfunctional assumptions about the world 2. Dysfunctional assumptions about the body 3. Dysfunctional assumptions about feelings	AN = 17 BN = 34 EDNOS = 28	28.59 (8.31)	100%
*Testable Assumptions Questionnaire for Eating Disorders Revised* *(TAQ-ED-R)*					
Dhokia et al. [25]	No	No factor analysis performed	Non-clinical = 128 AN = 25 BN = 47	25.60 (6.07) 27.10 (6.94) 28.30 (7.17)	100% 100% 100%
*Thoughts Questionnaire* *(TQ)*					
Cooper et al. [20]	Yes	PCA indicated a three-factor model: 1. Negative thoughts about eating 2. Positive thoughts about eating 3. Permissive thoughts	Study 1: Non-clinical = 258 Study 1: AN = 14 Study 1: Dieters = 17 Study 1: Control = 18 Study 2: BN = 12 Study 2: Dieters = 17 Study 2: Control = 20	25.70 (8.10) 31.10 (10.30) 29.30 (5.70) 29.80 (8.30) 27.50 (6.30) 28.10 (5.10) 28.00 (4.90)	100%
*Weight Influenced Self-Esteem Questionnaire* *(WISE-Q)*					
Trottier et al. [89]	Yes	EFA indicated a two-factor model: 1. ‘Generalised’ aspects of self-esteem 2. ‘Expected’ aspects of self-esteem	Study 1 Clinical ED = 184 Study 1 University = 248 Study 2 Clinical ED = 96	27.40 (8.60) 22.10 (4.04) 27.40 (8.40)	100% 100% 100%

AN, Anorexia Nervosa; AN (B/P), Anorexia Nervosa binge-purge subtype; BE, binge eating; BED, Binge Eating Disorder; BN, Bulimia Nervosa; CFA, confirmatory factor analysis; ED, eating disorder; EDNOS, eating disorder not otherwise specified; EFA, exploratory factor analysis; N/A, not applicable; PCA, principal components analysis; SD, standard deviation

? = Indicates no information found

^a^This study contained psychometric evaluation of both the MACQ and the BCDS

*Only subscales with an asterisk were considered for psychometric evaluation of its properties. Only subgroups within studies that met inclusion criteria for age were considered for psychometric evaluation of psychometric p0roperties

A total of 31 measures (or subscales) were identified that met inclusion criteria from the included studies (see Table 3 for summary of included measures). Nine of the included measures were subscales, and nine were a short-form or revised version of an original measure. These measures were the Beliefs About Appearance Questionnaire (BASS), Bulimic Automatic Thoughts Test (BATT), Body Checking Cognitions Scale (BCCS), Bulimia Cognitive Distortions Scale (BCDS), Bulimic Thoughts Questionnaire (BTQ), Eating Beliefs Questionnaire (EBQ), Eating Beliefs Questionnaire-18 (EBQ-18), Eating Disorder Beliefs Questionnaire (EDBQ, Eating Disorder Core Beliefs Questionnaire (ED-CBQ), Eating Disorder Core Beliefs Questionnaire Revised (ED-CBQ-R), Eating Disorder Inventory Body Dissatisfaction subscale (EDI [BD]), Eating Disorder Inventory II Body Dissatisfaction subscale (EDI-II [BD]), Eating Disorder Inventory III Body Dissatisfaction subscale (EDI-III [BD]), Eating Expectancy Inventory (EEI), Functions of Binge Eating Scale (FBES), Irrational Food Beliefs Scale (IFBS), Interpersonal Outcome Expectancy for Thinness scale (IOET), Mizes Anorectic Cognitions Questionnaire (MACQ), Mizes Anorectic Cognitions Questionnaire Brief (MACQ-B), Mizes Anorectic Cognitions Questionnaire Revised (MACQ-R), Muscle Dysmorphia Inventory Drive for Size subscale (MDDI [DS]), Perceived Benefits of Thinness Scale (PBTS), Sociocultural Attitudes Towards Appearance Questionnaire 4 Internalisation Thin subscale (SATAQ-4 [IT]), Sociocultural Attitudes Towards Appearance Questionnaire 4 Revised Internalisation Thin subscale (SATAQ-4R [IT]), Stirling Eating Disorders Scale Anorexic Dietary Cognitions subscale (SEDS [ADC]), Stirling Eating Disorders Scale Bulimic Dietary Cognitions subscale (SEDS [BDC]), Stirling Eating Disorders Scale Low Self-esteem subscale (SEDS [LSE]), Testable Assumptions Questionnaire for Eating Disorders (TAQ-ED), Testable Assumptions Questionnaire for Eating Disorders Revised (TAQ-ED-R), Thoughts Questionnaire (TQ), and the Weight Influenced Self-Esteem Questionnaire (WISE-Q).

**Table 3:** Description of measure

Measure	Construct/goal	Number of items	Response categories	Subscales/factors
BAAS	Dysfunctional attitudes about appearance	20	5-point Likert scale (0 = Not at all, 4 = Extremely)	1. Dysfunctional attitudes about appearance hypothesised to be unique to eating disorders
BATT	Automatic thoughts associated with bulimia	20	5-point Likert scale (1 = Not at all, 5 = all the time)	1. Automatic thoughts characterised by cognitive distortions associated with bulimia nervosa
BCCS	Cognitions underlying body checking behaviours	19	5-point Likert scale (1 = Never, 5 = Very often)	1. Objective verification beliefs 2. Reassurance beliefs 3. Safety beliefs 4. Body control beliefs
BCDS	Irrational beliefs associated with BN	25	5-point Likert scale (1 = strongly disagree, 5 = strongly agree)	1. Cognitive distortions associated with automatic eating behaviours 2. Cognitive distortions associated with physical appearance
BTQ	Cognitions related to bulimia	20	5-point Likert scale (1 = not at all, 5 = all the time)	1. Self-schema related to weight 2. Self-efficacy expectations 3. Salient beliefs
EBQ	Beliefs about binge eating	16	5-point Likert scale (1 = strongly disagree, 5 = strongly agree)	1. Positive beliefs 0about eating 2. Negative Beliefs about eating
EBQ-18	Beliefs about binge eating	18	5-point Likert scale (1 = strongly disagree, 5 = strongly agree)	1. Positive beliefs about eating 2. Negative beliefs about eating 3. Permissive beliefs about eating
EDBQ	Assumptions and beliefs associated with eating disorders	32	Visual analogue scale 1–100 (Never to Always)	1. Negative self-beliefs 2. Weight and shape as a means of acceptance by others 3. Weight and shape as a means to self-acceptance 4. Control overeating
ED-CBQ	Core beliefs associated with eating disorders	40	7-point Likert scale (1 = feels very much untrue, 7 = feels very much true)	1. Self-loathing 2. Unassertive/inhibited 3. High standards for self 4. Demanding/in need of help and support 5. Abandoned/deprived
ED-CBQ-R	Core beliefs associated with eating disorders	15	7-point Likert scale (1 = feels very much untrue, 7 = feels very much true)	1. Self-loathing 2. Unassertive/inhibited 3. Demanding/in need of help and support 4. Abandoned/deprived
EDI (BD)*	Body dissatisfaction in anorexia and bulimia nervosa	9	6-point Likert scale (1 = Never, 6 = Always)	1. Body dissatisfaction
EDI-II (BD)*	Body dissatisfaction in anorexia and bulimia nervosa	9	6-point Likert scale (1 = Never, 6 = Always)	1. Body dissatisfaction
EDI-III (BD)*	Body dissatisfaction in eating disorders	9–10	6-point Likert scale (1 = Never, 6 = Always)	1. Body dissatisfaction
EEI	Expectations related to eating	34	7-point Likert scale (1 = Completely disagree, 7 = Completely agree)	1. Eating helps manage negative affect 2. Eating is pleasurable and useful as a reward 3. Eating leads to feeling out of control 4. Eating enhances cognitive competence 5. Eating alleviates boredom
FBES	Functions of binge eating	46	5-point Likert scale (1 = Strongly disagree, 5 = Strongly agree)	1. Self-protection 2. Compensatory eating 3. Hedonic hunger 4. Emotional regulation 5. Control 6. Self-punishment 7. Emotion expression 8. Numbness/dissociation
IFBS	Irrational beliefs relating to food and eating	57	4-point Likert scale (1 = Strongly disagree, 4 = Strongly agree)	1. Irrational beliefs about food and eating 2. Rational beliefs about food and eating
IOET	Interpersonal outcome expectancies for being thin	8	7-point Likert scale (1 = strongly disagree, 5 = strongly agree)	1. Positive interpersonal outcome expectancies for being thin
MACQ	Cognitive distortions in anorexia and bulimia	33	5-point Likert scale (1 = strongly disagree, 5 = strongly agree)	1. Rigid weight and eating regulation 2. Weight and eating behaviour as the basis of approval from others 3. Self-esteem based off excessive self-control
MACQ-B	Cognitive distortions in anorexia and bulimia	12	5-point Likert scale (1 = strongly disagree, 5 = strongly agree)	1. Self-control and self esteem 2. Weight and approval 3. Rigid weight regulation and fear of weight gain
MACQ-R	Cognitive distortions in anorexia and bulimia	25	5-point Likert scale (1 = strongly disagree, 5 = strongly agree)	1. Self-control and self esteem 2. Weight and approval 3. Rigid weight regulation and fear of weight gain
MDDI (DS)*	Body dissatisfaction and drive for size	5	5-point Likert scale (1 = Never, 5 = Always)	1. Drive for size
PBTS	Beliefs about perceived benefits of thinness	12	6-point Likert scale (1 = No chance, 6 = Certain to happen)	1. Beliefs about perceived benefits of thinness
SATAQ-4 (IT)*	Internalization of attitudes relating to desiring thinness or low body fat	4	5-point Likert scale (1 = Definitely disagree, 5 = Definitely agree)	1. Internalisation—Thin/Low Body fat
SATAQ-4R (IT)*	Internalization of attitudes relating to desiring thinness or low body fat	4	5-point Likert scale (1 = Always/Strongly agree, 5 = Never/Strongly disagree)	1. Internalisation—Thin/Low Body fat
SEDS (ADC)*	Anorexic dietary cognitions	10	Dichotomous categorical (True/False)	1. Anorexic dietary cognitions
SEDS (BDC)*	Bulimic dietary cognitions	10	Dichotomous categorical (True/False)	1. Bulimic dietary cognitions
SEDS (LSE)*	Low self-esteem beliefs	10	Dichotomous categorical (True/False)	1. Low self-esteem beliefs
TAQ-ED	Eating disorder related dysfunctional assumptions	12	5-point Likert scale (1 = do not agree, 5 = totally agree)	1. Dysfunctional assumptions about the world 2. Dysfunctional assumptions about the body 3. Dysfunctional assumptions about feelings
TAQ-ED-R	Eating disorder related dysfunctional assumptions	20	5-point Likert scale (1 = do not agree, 5 = totally agree)	1. Dysfunctional assumptions about the world 2. Dysfunctional assumptions about the body 3. Dysfunctional assumptions about feelings
TQ	Automatic thoughts related to eating disorders	26	Likert scale 1–100 (1 = I do not usually believe this at all, 100 = I am usually completely convinced that this is true)	1. Negative thoughts about eating 2. Positive thoughts about eating 3. Permissive thoughts
WISE-Q	Influence of negative perception about body weight and/or shape on self-esteem	22	5-point Likert scale (0 = Not at all, 4 = Extremely)	1. ‘Generalised’ aspects of self-esteem (i.e., social, personality and performance domains) 2. ‘Expected’ aspects of self-esteem (i.e., appearance, self-control, etc.)

BAAS, Beliefs About Appearance Questionnaire; BATT, Bulimic Automatic Thoughts Test; BCCS, Body Checking Cognitions Scale; BCDS, Bulimia Cognitive Distortions Scale; BTQ, Bulimic Thoughts Questionnaire; EBQ, Eating Beliefs Questionnaire; EBQ-18, Eating Beliefs Questionnaire 18; EDBQ, Eating Disorder Beliefs Questionnaire; ED-CBQ, Eating Disorder Core Beliefs Questionnaire; ED-CBQ-R, Eating Disorder Core Beliefs Questionnaire Revised; EDI (BD), Eating Disorder Inventory Body Dissatisfaction subscale; EDI-II (BD), Eating Disorder Inventory II Body Dissatisfaction subscale; EDI-III (BD), Eating Disorder Inventory III Body Dissatisfaction subscale; EEI, Eating Expectancy Inventory; FBES, Functions of Binge Eating Scale; IFBS, Irrational Food Beliefs Scale; IOET, Interpersonal Outcome Expectancy for Thinness Scale; MACQ, Mizes Anorectic Cognitions Questionnaire; MACQ-B, Mizes Anorectic Cognitions Questionnaire Brief; MACQ-R, Mizes Anorectic Cognitions Questionnaire Revised; MDDI (DS), Muscle Dysmorphia Inventory Drive for Size subscale; PBTS, Perceived Benefits of Thinness Scale; SATAQ-4 (IT), Sociocultural Attitudes Towards Appearance Questionnaire 4 Internalisation Thin subscale; SATAQ-4R (IT), Sociocultural Attitudes Towards Appearance Questionnaire 4 Revised Internalisation Thin subscale; SEDS (ADC), Stirling Eating Disorders Scale Anorexic Dietary Cognitions subscale; SEDS (BDC), Stirling Eating Disorders Scale Bulimic Dietary Cognitions subscale; SEDS (LSE), Stirling Eating Disorders Scale Low Self-esteem subscale; TAQ-ED, Testable Assumptions Questionnaire for Eating Disorders; TAQ-ED-R, Testable Assumptions Questionnaire for Eating Disorders Revised; TQ, Thoughts Questionnaire; WISE-Q, Weight Influenced Self-Esteem Questionnaire

*Included measure is a subscale

The cognitive focus of each measure was described using four broad categories: (1) negative thoughts about food and eating, (2) negative thoughts about weight, shape, or body image, (3) self-referent beliefs, and (4) meta-cognitive beliefs (see Table 4). These categories were formed by examining the content of included measures and identifying the main areas of cognitive content and the types of cognitions assessed. Altogether, 15 measures assessed negative thoughts about food and eating, 20 assessed negative thoughts about weight, shape or body image, nine assessed self-referent beliefs, and nine assessed meta-cognitive beliefs. The BCDS, TAQ-ED, and TAQ-ED-R considered all four categories of cognitive focus.

**Table 4:** Cognitive focus of included measures

Measure	Cognitive focus			
	Negative thoughts: food and eating	Negative thoughts: weight, shape, body image	Self-referent beliefs	Meta-cognitive beliefs
Beliefs About Appearance Questionnaire (BAAS)	✓	✓		
Bulimic Automatic Thoughts Test (BATT)	✓	✓		
Body Checking Cognitions Scale (BCCS)		✓		
Bulimia Cognitive Distortions Scale (BCDS)	✓	✓	✓	✓
Bulimic Thoughts Questionnaire (BTQ)	✓	✓	✓	
Eating Beliefs Questionnaire (EBQ)	✓			✓
Eating Beliefs Questionnaire 18 (EBQ-18)	✓			✓
Eating Disorder Beliefs Questionnaire (EDBQ)			✓	✓
Eating Disorder Core Beliefs Questionnaire (ED-CBQ)			✓	
Eating Disorder Core Beliefs Questionnaire Revised (ED-CBQ-R)			✓	
Eating Disorder Inventory—Body Dissatisfaction Subscale (EDI [BD])		✓		
Eating Disorder Inventory II—Body Dissatisfaction Subscale (EDI-II [BD])		✓		
Eating Disorder Inventory III—Body Dissatisfaction Subscale (EDI-III [BD])		✓		
Eating Expectancy Inventory (EEI)				✓
Functions of Binge Eating Scale (FBES)				✓
Irrational Food Beliefs Scale (IFBS)	✓			
Interpersonal Outcome Expectancy for Thinness Scale (IOET)		✓		
Mizes Anorectic Cognitions Questionnaire (MACQ)	✓	✓		
Mizes Anorectic Cognitions Questionnaire Brief (MACQ-B)	✓	✓		
Mizes Anorectic Cognitions Questionnaire Revised (MACQ-R)	✓	✓		
Muscle Dysmorphia Inventory—Drive for Size Subscale (MDDI [DS])		✓		
Perceived Benefits of Thinness Scale (PBTS)		✓		
Sociocultural Attitudes Towards Appearance Questionnaire 4—Internalisation—Thin Subscale (SATAQ-4 [IT])		✓		
Sociocultural Attitudes Towards Appearance Questionnaire 4 Revised—Internalisation—Thin Subscale (SATAQ-4R [IT])		✓		
Stirling Eating Disorders Scale—Anorexic Dietary Cognitions (SEDS [ADC])	✓			
Stirling Eating Disorders Scale—Bulimic Dietary Cognitions (SEDS [BDC])	✓			
Stirling Eating Disorders Scale—Low Self-esteem (SEDS [LSE])			✓	
Testable Assumptions Questionnaire for Eating Disorders (TAQ-ED)	✓	✓	✓	✓
Testable Assumptions Questionnaire for Eating Disorders Revised (TAQ-ED-R)	✓	✓	✓	✓
Thoughts Questionnaire (TQ)	✓	✓		✓
Weight Influenced Self-Esteem Questionnaire (WISE-Q)		✓	✓	

### Assessment of psychometric properties

The psychometric properties of each included study were assessed using the criteria outlined by Terwee et al. [87]. This assessment was independently conducted by the same two reviewers who screened and assessed the studies for eligibility (AH and SB). Agreement between the reviewers for the criteria of adequacy was 94% (κ = 0.91). Consensus was reached to resolve discrepancies between the two assessors, and as such a third reviewer was not necessary. The summary ratings for each measure are displayed in Table 5.

**Table 5:** Quality analysis/ratings of psychometric properties

Measure	Content validity	Internal consistency	Criterion validity	Construct validity	Reproducibility–agreement (test–retest)	Reproducibility–reliability	Responsiveness	Floor and ceiling effects	Interpretability
BAAS	+	−	0	+	?	0	0	0	?
BATT	+	?	0	+	0	0	0	0	+
BCCS	+	+	0	+	0	0	0	0	?
BCDS	+	−	0	+	0	0	0	0	?
BTQ	+	?	0	0	0	0	0	0	?
EBQ	?	+	0	+	+	0	+	0	+
EBQ-18	?	+	?	+	+	+	+	0	+
EDBQ	+	+	?	+	−	0	0	0	?
ED-CBQ	+	−	0	+	0	0	0	0	?
ED-CBQ-R	+	+	0	+	0	0	−	−	+
EDI (BD)	?	?	?	+	?	0	0	0	+
EDI-II (BD)	?	?	?	+	?	0	0	0	?
EDI-III (BD)	?	+	?	+	+	+	0	+	?
EEI	+	−	0	+	0	0	0	0	?
FBES	?	+	0	+	0	0	0	0	0
IFBS	+	?	0	+	0	0	0	0	+
IOET	+	−	0	+	+	0	0	0	?
MACQ	−	+	0	+	+	0	0	0	?
MACQ-B	+	−	0	+	0	0	0	0	?
MACQ-R	+	+	0	+	0	0	0	0	+
MDDI (DS)	?	+	0	+	+	0	0	0	?
PBTS	−	+	0	+	+	0	0	0	0
SATAQ-4 (IT)	−	+	0	+	0	0	+	0	+
SATAQ-4R (IT)	−	+	0	+	?	0	0	0	+
SEDS (ADC)	+	?	0	+	?	0	0	0	+
SEDS (BDC)	+	?	0	+	?	0	0	0	+
SEDS (LSE)	+	?	0	+	?	0	0	0	+
TAQ-ED	−	−	0	+	0	0	0	0	?
TAQ-ED-R	−	−	0	?	0	0	0	0	?
TQ	−	+	−	+	0	0	0	0	+
WISE-Q	−	−	0	+	?	0	+	0	+

BAAS, Beliefs About Appearance Questionnaire; BATT, Bulimic Automatic Thoughts Test; BCCS, Body Checking Cognitions Scale; BCDS, Bulimia Cognitive Distortions Scale; BTQ, Bulimic Thoughts Questionnaire; EBQ, Eating Beliefs Questionnaire; EBQ-18, Eating Beliefs Questionnaire 18; EDBQ, Eating Disorder Beliefs Questionnaire; ED-CBQ, Eating Disorder Core Beliefs Questionnaire; ED-CBQ-R, Eating Disorder Core Beliefs Questionnaire Revised; EDI (BD), Eating Disorder Inventory Body Dissatisfaction subscale; EDI-II (BD), Eating Disorder Inventory II Body Dissatisfaction subscale; EDI-III (BD), Eating Disorder Inventory III Body Dissatisfaction subscale; EEI, Eating Expectancy Inventory; FBES, Functions of Binge Eating Scale; IFBS, Irrational Food Beliefs Scale; IOET, Interpersonal Outcome Expectancy for Thinness Scale; MACQ, Mizes Anorectic Cognitions Questionnaire; MACQ-B, Mizes Anorectic Cognitions Questionnaire Brief; MACQ-R, Mizes Anorectic Cognitions Questionnaire Revised; MDDI (DS), Muscle Dysmorphia Inventory Drive for Size subscale; PBTS, Perceived Benefits of Thinness Scale; SATAQ-4 (IT), Sociocultural Attitudes Towards Appearance Questionnaire 4 Internalisation Thin subscale; SATAQ-4R (IT), Sociocultural Attitudes Towards Appearance Questionnaire 4 Revised Internalisation Thin subscale; SEDS (ADC), Stirling Eating Disorders Scale Anorexic Dietary Cognitions subscale; SEDS (BDC), Stirling Eating Disorders Scale Bulimic Dietary Cognitions subscale; SEDS (LSE), Stirling Eating Disorders Scale Low Self-esteem subscale; TAQ-ED, Testable Assumptions Questionnaire for Eating Disorders; TAQ-ED-R, Testable Assumptions Questionnaire for Eating Disorders Revised; TQ, Thoughts Questionnaire; WISE-Q, Weight Influenced Self-Esteem Questionnaire

#### Content validity

Content validity refers to the extent that the items of a measure are an accurate reflection of the construct of interest [87]. To have received a positive rating, studies (measure or subscale) describing the development of the measure were required to provide a clear description of the aim of the measure, concepts measured, target population and item selection. Further, they were required to have had experts and a relevant target population involved in item selection. Seventeen measures received a positive rating for this criterion, including the BASS, BATT, BCCS, BCDS, BTQ, EDBQ, ED-CBQ, ED-CBQ-R, EEI, IFBS, IOET, MACQ-B, MACQ-R, SEDS (ADC) subscale, SEDS (BDC) subscale, and the SEDS (LSE) subscale. These findings indicated that these measures (or subscales) demonstrated adequate content validity.

#### Internal consistency

Internal consistency refers to the extent to which items in a measure are correlated, and thus assess the same construct [87]. To have received a positive rating, studies must have reported a Cronbach’s alpha of adequate magnitude for the measure (or subscale) and performed a factor analysis using an adequate sample size. When assessing internal consistency for subscales, factor analysis was considered if it was performed solely on the subscale or on the full scale if it tested the included subscale as a factor. Fourteen measures received a positive rating for this criterion. These findings indicated that BATT, EBQ, EBQ-18, EDBQ, ED-CBQ-R, EDI-III (BD) subscale, FBES, MACQ, MACQ-R, MDDI (DS) subscale, PBTS, SATAQ-4 (IT) subscale, SATAQ-4R (IT) subscale, and the TQ have demonstrated adequate internal consistency.

#### Criterion validity

According to the Terwee et al. [87] criteria, criterion validity is determined by comparison to a ‘gold-standard’ instrument, ensuring the new measure is theoretically related to a well-established measure. Given that we imposed no limits on studies and measures included by time period, and that there is no suitable or widely agreed upon gold standard for assessing ED cognitions, if the study assessed for criterion validity as per Terwee et al. [87] definition, we allowed each study and its authors to provide their own justification or a convincing argument for their definition of a gold-standard. While the term ‘criterion validity’ was indeed used in some studies, the authors were, in fact, referring to other forms of validity (e.g., content validity or construct validity). No measures received a positive rating, most received a ‘no information available’ rating. Five measures (EBQ-18, EDBQ, EDI [BD] subscale, EDI-II [BD] subscale, EDI-III [BD] subscale) received an indeterminate rating for a lack of convincing argument for the gold standard measure utilised for comparison, and one (TQ) received a negative rating as the correlation with the proposed gold standard was < 0.07.

#### Construct validity

Construct validity refers to the degree to which scores on a particular questionnaire relate (or do not relate) to other measures in a manner that is consistent with theoretically derived hypotheses concerning the concepts that are being measured [87]. To have received a positive rating, studies were required to provide clear predictions regarding their hypotheses with 75% of the results in the expected direction (e.g., reporting the correlation between two measures in the expected direction)’. Almost all measures received a positive rating for this criterion, indicating the vast majority possessed adequate construct validity. Only the TAQ-ED-R received an indeterminate rating, and the BTQ received a ‘no information available’ rating.

#### Reproducibility: agreement (test–retest)

Agreement refers to the extent to which scores on a measure remain stable over time. According to Terwee et al. [87] adequate agreement is demonstrated when the absolute measurement error is smaller than the Minimally Important Change (MIC) factor. However, MIC was not defined or utilised in any of the studies evaluated in this review. Therefore, we utilised the criterion for agreement previously used by Burton et al. [9], and Zuccala et al. [101], defining adequate agreement as a test–retest reliability of *r* > 0.70. To have received a positive rating for this criterion, the means and standard deviations must have been presented at both time points. Seven measures received a positive rating for this criterion, indicating the EBQ, EBQ-18, EDI-III (BD) subscale, IOET, MACQ, MDDI (DS) subscale, and the PBTS possessed adequate test–retest agreement.

#### Reproducibility: reliability

Reproducibility reliability refers to the extent to which individuals can be distinguished from each other [87]. To have received a positive rating measures needed to provide an intraclass correlation or weighted Kappa > 0.70 to test this. The EBQ-18 and the EDI-III (BD) subscale were the only measures found to demonstrate adequate reliability. All other measures all received a ‘no information available’ rating.

#### Responsiveness

Responsiveness refers to the ability of a measure to detect clinically important changes over time or following an intervention [87]. To have received a positive rating, the studies must have outlined a treatment program and the longitudinal expected changes, and 75% of results must have been in the expected direction. Alternatively, measures had to demonstrate a responsiveness ratio of > 1.96 or an area under the curve > 0.70. Only the EBQ, EBQ-18, SATAQ-4 (IT) subscale, and WISE-Q demonstrated adequate responsiveness.

#### Floor and ceiling effects

According to Terwee et al. [87], floor and ceiling effects have occurred when > 15% of participants achieve the highest or lowest possible score on a measure (or subscale). Only the EDI-III (BD) subscale demonstrated a positive rating for this criterion, for reporting information demonstrating a lack of floor and ceiling effects. The ED-CBQ-R demonstrated a floor effect on one of its subscales, consequently receiving a negative rating. All other measures all received a ‘no information available’ rating.

#### Interpretability

Interpretability refers to the extent to which qualitative meaning can be given to quantitative scores [87]. To have received a positive rating, a measure must have presented means and standard deviation scores for at least four relevant subgroups within one study (in isolation). As per Terwee et al.’s suggestion, subgroups stratified by demographic variables (e.g., age, gender) may be included as subgroups. Further, as in Zuccala et al. [101], this criterion was modified such that a minimal important change (MIC) was not required to have received a positive rating, as no studies reported a MIC. Findings indicated that 14 measures (or subscales) possessed adequate interpretability, including the BATT, EBQ, EBQ-18, ED-CBQ-R, EDI (BD) subscale, IFBS, MACQ-R, SATAQ-R (IT) subscale, SATAQ-4R (IT) subscale, SEDS (ADC) subscale, SEDS (BDC) subscale, SEDS (LSE) subscale, TQ, and the WISE-Q. As Modini et al. [59] and Zuccala et al. [101] have previously noted, it is important to note that if considering an accumulation of subgroups between studies (not within one study in isolation), it is likely that more measures would have met this criterion.

## Discussion

Prioritising the assessment of ED cognitions is essential for monitoring key factors contributing to the development and maintenance of EDs. It is essential that self-report measures possess adequate psychometric properties to increase their validity, reliability and utility for clinical research and practice. This systematic review aimed to evaluate the psychometric properties of existing self-report measures of ED cognitions using the Terwee et al. [87] criteria of adequacy for measurement properties, to conduct quality assessment.

This review identified 59 studies that evaluated the psychometric properties of 31 self-report measures (or subscales) that assess ED cognitions. The type of cognitive focus examined most across these measures was negative thoughts about weight, shape or body image, followed then by negative thoughts about food and eating. Self-referent and meta-cognitive beliefs were types of cognition assessed to a lesser extent across the included measures. This suggests that the majority of self-report measures specifically developed to assess ED cognitions have primarily been focused on the ‘thought’ level, where fewer have been developed to examine longstanding or engrained beliefs sets, including conditional and unconditional assumptions and beliefs.

With respect to the psychometric properties of included measures, no measure (or subscale) received positive ratings across all categories. The criterion that received the greatest number of positive ratings across all measures was construct validity. This suggests firstly that there is a propensity for studies to examine construct validity, as compared to other psychometric properties which are less routinely considered, and also of a general trend that existing measures assessing ED cognitions show demonstrate strong evidence for construct validity. This is compared to other criterion categories, which findings demonstrate are evaluated and considered less overall. Examples include criterion validity, reproducibility, reliability, responsiveness and floor and ceiling effects, where the majority of measures had no information found on the assessment of these properties. Interestingly, in terms of content validity, most studies and measures indeed clearly described the concepts measured, the intended target population, also involving experts in item selection. However, all measures with an indeterminate rating were characterised by a lack of clarity about whether and how the target population had been involved in item selection, and those with a negative rating failed to describe any involvement of the target population in item selection. It is clearly important to have items reflecting cognitive constructs that have been acknowledged as relevant by those populations whose cognitions the measures are attempting to describe and assess.

In relation to the four broad categories of cognitive focuses identified, some specific observations and subsequent recommendations can be made. Whilst attempting to assess negative thoughts relating to food and eating, the EBQ-18 currently possesses the most evidence for its validity, reliability and utility, compared to other measures assessing similar content. Relatedly, of measures that assess negative thoughts related to weight, shape and body image, the EDI-III (BD) subscale and the SATAQ-4 (IT) subscale have the most psychometric support. However, it is important to note these measures both focus on body dissatisfaction that implies desire for a thinner body, as opposed to, for example, a more muscular body. Thus, even within the categories identified, it remains important to consider the construct of interest when selecting and utilising a cognitive ED measure. With respect to self-referent beliefs, the ED-CBQ-R currently has the most evidence for its validity, reliability and utility. The EBQ-18 has the most empirical support for its psychometric properties of measures that assess meta-cognitive beliefs related to EDs.

An overall pattern also emerged indicating that newer, revised versions of an original measure demonstrated a greater number of positive ratings, as evidenced by the EBQ-18, ED-CBQ-R, EDI-III (BD), and the MACQ-R. The results of this systematic review also suggest that the EBQ, EBQ-18, and EDI-III (BD) have the most evidence in support of their psychometric properties. However, a strict conclusion cannot be drawn that these measures are objectively superior, as not all measurement properties are necessarily equally important [87]. As previously noted [101], different measures may be utilised for different purposes, and certain psychometric properties may have varied importance under diverse circumstances. For example, for the purposes of a clinical trial, adequate responsiveness might be of greatest importance, whereas in a prevalence study aiming to identify the presence or absence of various cognitions, choosing a measure with greater breadth may be more beneficial. Moreover, conclusions cannot be made as to the superiority of a specific measure, as this review considers different types of ED cognitions. The included self-report measures of ED cognitions examine different constructs (e.g., body dissatisfaction versus meta-cognitive beliefs about eating), and types of cognitions (e.g., automatic thoughts versus unconditional beliefs). Thus, we recommend those wishing to utilise measures with more evidence for their validity, reliability and utility, should also consider the construct of interest, its intended usage, and the type of cognition to be assessed.

This systematic review had several notable strengths, including the standardised and systematic approach applied through its utilisation of a previously validated tool, widely utilised in previous systematic reviews evaluating psychometric properties of self-report measures [9, 59, 84, 101]. This review also demonstrated evidence of good interrater reliability at several stages of the review process. Additionally, the present review included all versions of measures in included studies, regardless of whether the measure was an original, revised or short version of a self-report measure. This facilitated a comprehensive summary and allowed for comparison of the state of evidence for each questionnaire version.

It is also important to note several limitations, the first of which is the stringent nature of the appraisal of quality tool utilised in the present study. The criteria used may have meant that some measures received either an intermediate or negative rating in accordance with strict standards for appraisal of adequacy, when using less exacting criteria might have resulted in a more positive rating. For example, we adhered to the strict Cronbach’s alpha cut-off in assessing internal consistency, where a few studies presented alphas that were only marginally above or below the range given in order to give a positive rating. The authors attempted to address this in several instances whilst still maintaining the intended rigor, by providing some leniency across ratings as described previously, and in alignment with carefully considered modifications that have also been employed in previous systematic reviews [9, 101]. For example, in test–retest reliability, in consenting to studies measuring reliability using more frequently utilised statistical methods. Further, authors were somewhat lenient in terms of content validity where body image measures were concerned, as the requirement for ‘involving the target population in item selection’ meant that the target population did not necessarily need to be a clinical ED population, but simply those presenting with varied body image concerns. Altogether, it was considered necessary and beneficial to continue to maintain this standard in order to recommend usage of psychometrically sound measures, and importantly to provide researchers incentive to continue to improve quality of existing and future assessment tools, and by extension, improve quality of empirical evidence more generally.

Additionally, it is important to consider the strict nature of our inclusion and exclusion criteria alongside the evidence presented. A limitation of this review is that only articles utilising an adult population and those utilising a non-English speaking population were considered for inclusion. As such, some measures or studies in support of certain psychometric properties might have been excluded in accordance with these criteria. Ultimately, studies utilising non-English speaking populations or measures not administered in English were out of the scope of this review. The authors also considered the importance of decreasing some heterogeneity in the evidence evaluated in the context of this systematic review [51]. This was relevant also to the exclusion of studies utilising child, purely adolescent, or mixed populations. As a function of differences in ED symptomatology across developmental stages [23, 50], it was important to consider potential differences in ED cognitions between adult and child or adolescent populations, and that some assessment tools may be valid and reliable in one population and not another.

Finally, the stringency of our criteria regarding measures being developed specifically and solely to measure ED cognitions, meant some more commonly utilised ED measures were not included in this systematic review. This included the EDE-Q [26] concerns subscales and versions of the Eating Attitudes Test (EAT-40 [34], EAT-40 [35]). Examination of their intention during development and careful scrutiny at the item level reflected that they captured other symptomatology, including emotional, behavioural symptoms, and items measuring functional impairment. These exclusions may impact the clinical utility of this review, due to the frequency of use of these measures for clinical and diagnostic purposes [42]. However, we also considered that the psychometric properties of these two measures have been assessed and systematically reviewed elsewhere [5, 45, 67]. Another commonly utilised cognitive measure not considered for inclusion was the Young Schema Questionnaire (YSQ-S3) [100]. Although useful in capturing core cognitive constructs, it was not developed specifically to capture ED cognitions, which was the focus of this systematic review. Finally, although this study included the EDI-III (BD), previous systematic reviews have considered the EDI-III (BD) to assess both evaluative and affective components of body image [48]. Due to this discrepancy, we recommend findings be interpreted with caution.

The results of this systematic review suggest several areas of improvement for future research. Primarily, our findings do not necessarily indicate that measures lacking adequate evidence should not be utilised, but primarily highlights where there is either an absence of psychometric support, there is clear suggestion for future researchers to focus efforts on improving evidence for the validity, reliability and utility of these measures for the populations considered. There is perhaps greater need to consider the responsiveness and clinical utility of ED cognitive measures, as well as less widely considered measurement properties, such as criterion validity and examining floor and ceiling effects.

When examining the cognitive focus, content and types of cognitions of included measures, it is clear that most tend to consider only AN, BN, BED, and assessment of body image concerns. There is certainly space to consider the development and availability of tools to assess cognitions in comparatively less well researched EDs, or those with rapidly increasing clinical focus. This includes disorders such as Avoidant Restrictive Food Intake Disorder (ARFID) and orthorexia nervosa, which currently only have symptomatic and behavioural inventories, and tools available to support diagnosis. Finally, whilst examining populations most frequently utilised in included studies, it is clear that research needs to continue to prioritise inclusion of more male and gender-diverse samples. This is particularly critical due to growing evidence outlining the increased and substantial ED risk in gender-diverse individuals [37, 39, 81].

Altogether, the present study was the first to provide a systematic review of self-report measures of ED cognitions, and valuable information about the existing, relevant evidence for their psychometric properties. This information provided a basis for the future selection of valid, reliable and clinically useful tools for measuring a variety of ED cognitions. Although no measure appeared to possess adequate evidence across all nine measurement criteria, several provided a good amount of evidence in support of their reliability, validity and utility. Ultimately, comprehensive information was provided to support future selection of measure of ED cognitions dependent on the specific aims of research and/or treatment. It is hoped that the findings of the present review assist both researchers and clinicians alike in identifying, evaluating and comparing relevant measures for use in identifying and monitoring important treatment targets in clinical, sub-clinical or prodromal ED populations.

## Data Availability

The data using during the current review is available from the corresponding author on reasonable request.

## Abbreviations

AN: Anorexia Nervosa
AN (B/P): Anorexia Nervosa binge-purge subtype
APA: American Psychiatric Association
ARFID: Avoidant Restrictive Food Intake Disorder
AUC: Area under the curve
BAAS: Beliefs About Appearance Questionnaire
BATT: Bulimic Automatic Thoughts Test
BCCS: Body Checking Cognitions Scale
BCDS: Bulimia Cognitive Distortions Scale
BE: Binge eating
BED: Binge Eating Disorder
BN: Bulimia Nervosa
BTQ: Bulimic Thoughts Questionnaire
CBT-E: Cognitive Behavioural Therapy Enhanced
CFA: Confirmatory Factor Analysis
EAT: Eating Attitudes Test
EBQ: Eating Beliefs Questionnaire
EBQ-18: Eating Beliefs Questionnaire 18
ED: Eating Disorder
EDBQ: Eating Disorder Beliefs Questionnaire
ED-CBQ: Eating Disorders Core Beliefs Questionnaire
ED-CBQ-R: Eating Disorder Core Beliefs Questionnaire Revised
EDE-Q: Eating Disorder Examination Questionnaire
EDI (BD): Eating Disorder Inventory Body Dissatisfaction subscale
EDI-II (BD): Eating Disorder Inventory II Body Dissatisfaction subscale
EDI-III (BD): Eating Disorder Inventory III Body Dissatisfaction subscale
EDNOS: Eating disorder not otherwise specified
EEI: Eating Expectancy Inventory
EFA: Exploratory Factor Analysis
FBES: Functions of Binge Eating Scale
ICC: Intraclass correlation
IFBS: Irrational Food Beliefs Scale
IOET: Interpersonal Outcome Expectancy for Thinness Scale
LOA: Limits of agreement
MACQ: Mizes Anorectic Cognitions Questionnaire
MACQ-B: Mizes Anorectic Cognitions Questionnaire Brief
MACQ-R: Mizes Anorectic Cognitions Questionnaire Revised
MBDS: Male Body Dissatisfaction Scale
MDDI (DS): Muscle Dysmorphia Inventory Drive for Size subscale
MIC: Minimally Important Change
N/A: Not applicable
NAT: Negative automatic thoughts
OSFED: Other Specified Feeding and Eating Disorders
PBTS: Perceived Benefits of Thinness Scale
PCA: Principal components analysis
PRISMA: Preferred Reporting Items for Systematic Reviews and Meta-analyses
ROC: Receiver operating characteristic curve
RR: Responsiveness Ratio
SATAQ-4 (IT): Sociocultural Attitudes Towards Appearance Questionnaire 4 Internalisation Thin subscale
SATAQ-4R (IT): Sociocultural Attitudes Towards Appearance Questionnaire 4 Revised Internalisation Thin subscale
SD: Standard Deviation
SDC: Smallest detectable change
SEDS (ADC): Stirling Eating Disorders Scale Anorexic Dietary Cognitions subscale
SEDS (BDC): Stirling Eating Disorders Scale Bulimic Dietary Cognitions subscale
SEDS (LSE): Stirling Eating Disorders Scale Low Self-esteem subscale
TAQ-ED: Testable Assumptions Questionnaire for Eating Disorders
TAQ-ED-R: Testable Assumptions Questionnaire for Eating Disorders Revised
TQ: Thoughts Questionnaire
WISE-Q: Weight Influenced Self-Esteem Questionnaire
YSQ: Young Schema Questionnaire

## Footnotes

1 As an example, the Eating Disorder Examination Questionnaire (EDE-Q; [26] Eating, Weight and Shape Concerns subscales were each considered for inclusion during screening and study selection stage of the review. However, closer examination at the item level revealed each subscale contained items measuring behaviours, emotions, or other symptoms (e.g., functional impairment). As each subscale was not developed solely to assess cognitions (as per the inclusion criteria), studies examining the psychometric properties of the EDE-Q subscales were ultimately excluded from this review.
